# Application of algorithms based on improved YOLO in MRI image detection of brain tumors

**DOI:** 10.3389/fneur.2025.1646476

**Published:** 2025-09-26

**Authors:** Jinghui Chen, Tao Yang, Lianxin Xie, Lanlan Yang, Hongjia Zhao

**Affiliations:** ^1^The First Clinical Medical College, The Affiliated People’s Hospital of Fujian University of Traditional Chinese Medicine, Fuzhou, Fujian, China; ^2^The Affiliated People’s Hospital of Fujian University of Traditional Chinese Medicine, Fuzhou, Fujian, China

**Keywords:** artificial intelligence, brain tumor, magnetic resonance, YOLO11n, target detection

## Abstract

Brain tumors, characterized by irregular cell growth in the brain or surrounding tissues, encompass aggressive types like glioblastoma and more indolent forms such as meningiomas and pituitary tumors, often leading to increased intracranial pressure, neurological dysfunction, and low survival rates despite multimodal treatment. Early and precise identification of tumor subtypes in MRI images remains challenging due to image noise, heterogeneity, and morphological variability, limiting real-time clinical diagnostics. To address these issues, we propose an improved YOLO11n model for brain tumor detection, incorporating lightweight GhostConv modules for reduced redundancy, Online Convolutional Reparameterization (OREPA) in the C3k2 module for enhanced efficiency, and Efficient Multi-scale Attention (EMA) for better multiscale feature capture. Using 4,000 annotated MRI images from a public Kaggle dataset (glioma, meningioma, pituitary tumor, and no tumor), divided into training, validation, and test sets (8:1:1 ratio), the model was trained over 200 epochs and evaluated on internal and external sets. The optimized model achieved a mean average precision (mAP@50) of 97.2% and recall of 93.8%, surpassing the baseline YOLO11n by 2.1% in mAP@50 while reducing GFLOPS by 25% from 6.4 to 4.8, demonstrating superior accuracy, efficiency, and lightweight design for edge deployment. This approach not only facilitates rapid tumor localization and classification in clinical practice but also supports personalized treatment planning, offering extensible solutions for broader medical imaging applications and improved patient outcomes.

## 1 Introduction

Brain tumor is the irregular growth of cells in the brain or its surrounding tissues, brain tumor has strong proliferation, when it occupies a certain space, it will constantly compress the surrounding tissues, and then damage the central nervous system, threatening patients’ lives (1, 2). Glioblastoma is the most common and aggressive malignant brain tumor, with abnormal cell proliferation that can lead to increased intracranial pressure and progressive neurological dysfunction. Recent data shows that despite combined treatment with surgery, radiotherapy, and temozolomide, the five-year relative survival rate remains extremely low, at only approximately 5% (3). In this critical context, early and precise identification of tumor subtypes, including highly aggressive gliomas, benign but recurrent meningiomas, and pituitary tumors with insidious growths, is of decisive clinical significance for the development of personalized surgical protocols, precise planning of radiotherapy targets, and assessment of prognosis. Magnetic resonance imaging (MRI) has become an irreplaceable tool for the diagnosis of brain tumors by virtue of its multi-sequence imaging capability (e.g., T1-weighted, T2-weighted, FLAIR, and DWI sequences), sub-millimeter spatial resolution, and absence of ionizing radiation (4, 5). However, brain tumor MRI diagnosis also faces multiple challenges. At the level of image quality, the signal-to-noise ratio of images is significantly reduced due to patient autonomous motion, magnetic field inhomogeneity, and radiofrequency coil coupling noise. Morphologically, the three main types of tumors are highly heterogeneous, with gliomas often presenting as “butterfly-shaped” infiltrative growth with cystic necrosis in the white matter region, with blurred boundaries with the surrounding edema zone; meningiomas mostly present as homogeneously reinforced masses attached to the wide base of the dura mater, with a typical “rat-tail sign”; and pituitary tumors are hidden in the pyriform region, which are prone to wrapping around the carotid artery and compressing the optic crossroads (6, 7).

To overcome the diagnostic bottleneck, early approaches for brain tumor detection primarily relied on artificial feature engineering combined with machine learning classifiers. Kumar et al. extracted texture features using a multiscale wavelet transform with a grayscale covariance matrix and subsequently applied Support Vector Machines (SVMs) to classify gliomas, meningiomas, pituitary tumors, and normal brain tissues (8). Jareena et al. employed a Discrete Wavelet Transform (DWT) for noise reduction, followed by Principal Component Analysis (PCA) for dimensionality reduction, and then used a Random Forest classifier for tumor classification (9). Ginni et al. proposed a hybrid ensemble method integrating KNN, Random Forest, and Decision Tree classifiers. Their workflow first performed tumor segmentation using OTSU thresholding, then extracted low-dimensional features through Smooth Wavelet Transform (SWT), PCA, and Gray-Level Co-occurrence Matrix (GLCM), and finally combined the classifiers through majority voting to improve accuracy (10). Similarly, Basthikodi et al. extracted features by combining Histogram of Oriented Gradients (HOG) and Local Binary Patterns (LBP), and after PCA-based dimensionality reduction, employed SVMs for multiclass brain tumor classification, further demonstrating the effectiveness of traditional machine learning approaches in this task (11). Although traditional machine learning methods improved detection accuracy through preprocessing techniques such as resolution enhancement, contrast adjustment, and edge preservation, they rely heavily on manually engineered features. This dependence makes it difficult to capture complex information related to texture, edges, and internal structures, thereby limiting both accuracy and computational efficiency. With the rapid advancement of deep learning, especially in target detection, MRI-based brain tumor analysis has shifted toward convolutional neural networks (CNNs) as the dominant baseline models. For example, Ayadi et al. developed a customized multilayer CNN in which the initial layers used multi-scale convolution (3 × 3 and 5 × 5), followed by Batch Normalization, ReLU activation, and Dropout; two fully connected layers were then applied to output classification probabilities for three tumor types (12). Zahoor et al. proposed Res-BRNet, a CNN that integrates Regional and Residual modules. Their approach first extracted homogeneity and boundary features through a spatial block, then captured local and global texture differences via consecutive residual blocks, followed by a Global Average Pooling and Softmax layer for multiclass tumor classification (13). Previous studies have primarily focused on classification or segmentation tasks, whereas the present approach employs object detection to achieve both localization and classification, thereby better addressing the clinical need for precise tumor localization.

However, existing studies still fall short of meeting the clinical demand for real-time, high-precision brain tumor detection, as traditional deep learning models continue to face the trade-off between accuracy and efficiency. Although recent research has attempted to address these issues through CNN architecture optimizations (14, 15), achieving substantial lightweighting for real-time edge computing while maintaining or improving detection accuracy remains a major challenge. To bridge this gap, a lightweight brain tumor detection model with both high accuracy and efficiency is proposed. YOLO11n is adopted as the baseline framework owing to its advantages in real-time target detection, and three major enhancements are incorporated: (1) the GhostConv module to reduce redundant parameters, (2) the Online Convolutional Reparameterization (OREPA)-optimized C3k2 module to lower computational complexity (16), and (3) the Efficient Multiscale Attention (EMA) mechanism to strengthen multiscale feature representation. These improvements collectively aim to enhance detection accuracy and recall for complex MRI tumor features while significantly reducing computational cost, thereby improving feasibility for clinical deployment.

Deep learning-based object detection algorithms are generally categorized into one-stage [e.g., SSD (17), YOLO series (18, 19)] and two-stage approaches [e.g., R-CNN (20), Fast R-CNN (21), Faster R-CNN (22)]. Among them, the YOLO family has been widely applied in medical imaging due to its real-time performance, high accuracy, and end-to-end design. The latest YOLO11^1^ (23) integrates dynamic kernel convolution (C3k2 module) with a dual-label assignment strategy, offering improvements in both accuracy and speed while avoiding post-processing with non-maximum suppression. Nonetheless, limitations remain in brain tumor detection scenarios: convolutional stacking results in high parameter counts and computational cost, downsampling reduces spatial resolution, and high-intensity edema signals in T2-FLAIR sequences often obscure critical tumor features. Aiming at the above defects, this study proposes an improved brain tumor MRI classification and detection model based on the YOLO11n model. Firstly, the GhostConv module is used to reconstruct the feature extraction layer (24), and the redundant parameters are effectively compressed by 30% through a third-order process of primary feature generation, depth-separable linear transformation and cross-channel splicing. Secondly, the OREPA technique is embedded into the C3K2 module (25), which realizes the transformation of Bottleneck structure to single-layer inference by eliminating the batch normalization layer, introducing learnable linear scaling, and multi-branch equivalent fusion. Finally, the EMA mechanism is added to the Backbone key layer and the feature fusion network (26, 27), and the EMA mechanism obtains an efficient multiscale attention mechanism without dimensionality reduction by modifying the sequential processing method of the Coordinate Attention (CA) mechanism.

In summary, this study introduces an improved YOLO11n-based framework for brain tumor MRI detection. The proposed model integrates GhostConv, OREPA, and EMA modules to simultaneously achieve higher detection accuracy, enhanced recall, and reduced computational complexity. The primary objective is to provide a lightweight yet precise detection model that can support real-time clinical diagnosis and facilitate practical deployment in healthcare environments.

## 2 Improved method

### 2.1 Original YOLO model

**Figure 1:**
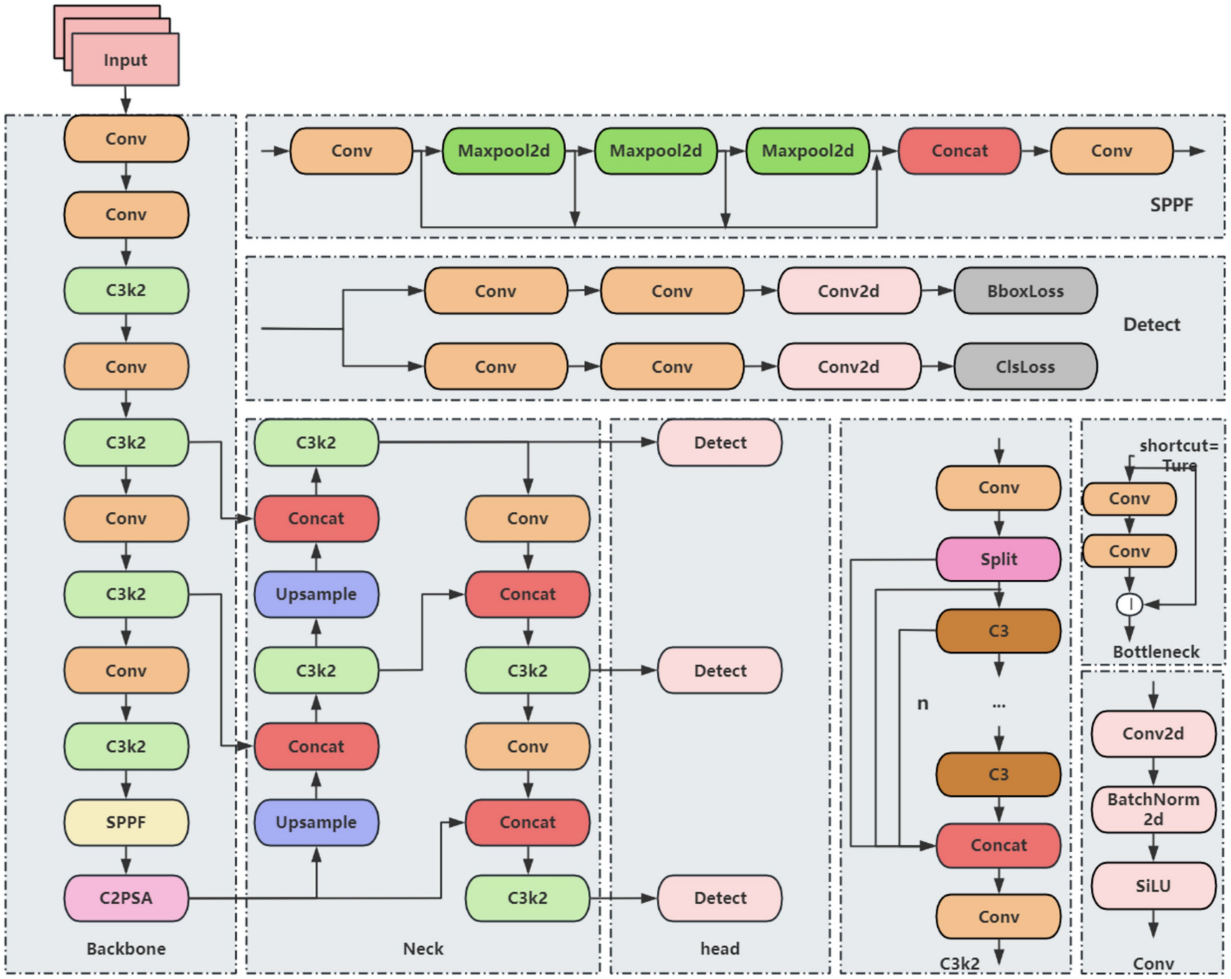
YOLO11 basic network structure.

The YOLO (You Only Look Once) series is a classic set of algorithms in the field of object detection, known for real-time performance, high accuracy, and ease of use. YOLO11 represents a mainstream object detection framework and includes five variants: YOLO11n, YOLO11s, YOLO11m, YOLO11l, and YOLO11x. Its architecture is primarily composed of three components: Backbone, Neck, and Head. Compared with its predecessors, such as YOLOv8 and YOLOv10, YOLO11 introduces several key innovations. The C3k2 module, an optimized variant of the CSP structure, improves computational efficiency by segmenting feature maps and enhancing information flow through small-kernel convolutions. This module can be configured to connect in tandem or be degraded to the C2f module of YOLOv8, indirectly increasing precision by deepening feature representations. A newly added module, C2PSA, integrates the C2f structure with a Partial Self-Attention (PSA) mechanism. After feature splitting via a 1 × 1 convolution, a portion of the features is forwarded directly, while the remaining features are processed by the PSA using multi-scale convolutions and weighted by an SE module. Finally, channels are point-wise weighted through a Softmax operation, enhancing attention to critical features and improving the detection of multi-scale objects in complex scenes. Additionally, the detection head adopts depthwise separable convolutions, inspired by YOLOv10, which reduces redundant computations and further improves inference efficiency. Despite these improvements, the YOLO11 baseline model still faces limitations, including high computational costs in the backbone network, insufficient feature extraction leading to partial loss of target details, and challenges in capturing small objects in low-resolution feature maps. These limitations constrain overall detection accuracy. Based on these considerations, YOLO11 was selected as the baseline model for further improvement. Its network architecture is illustrated in Figure 1.

### 2.2 Improved YOLO algorithm

**Figure 2:**
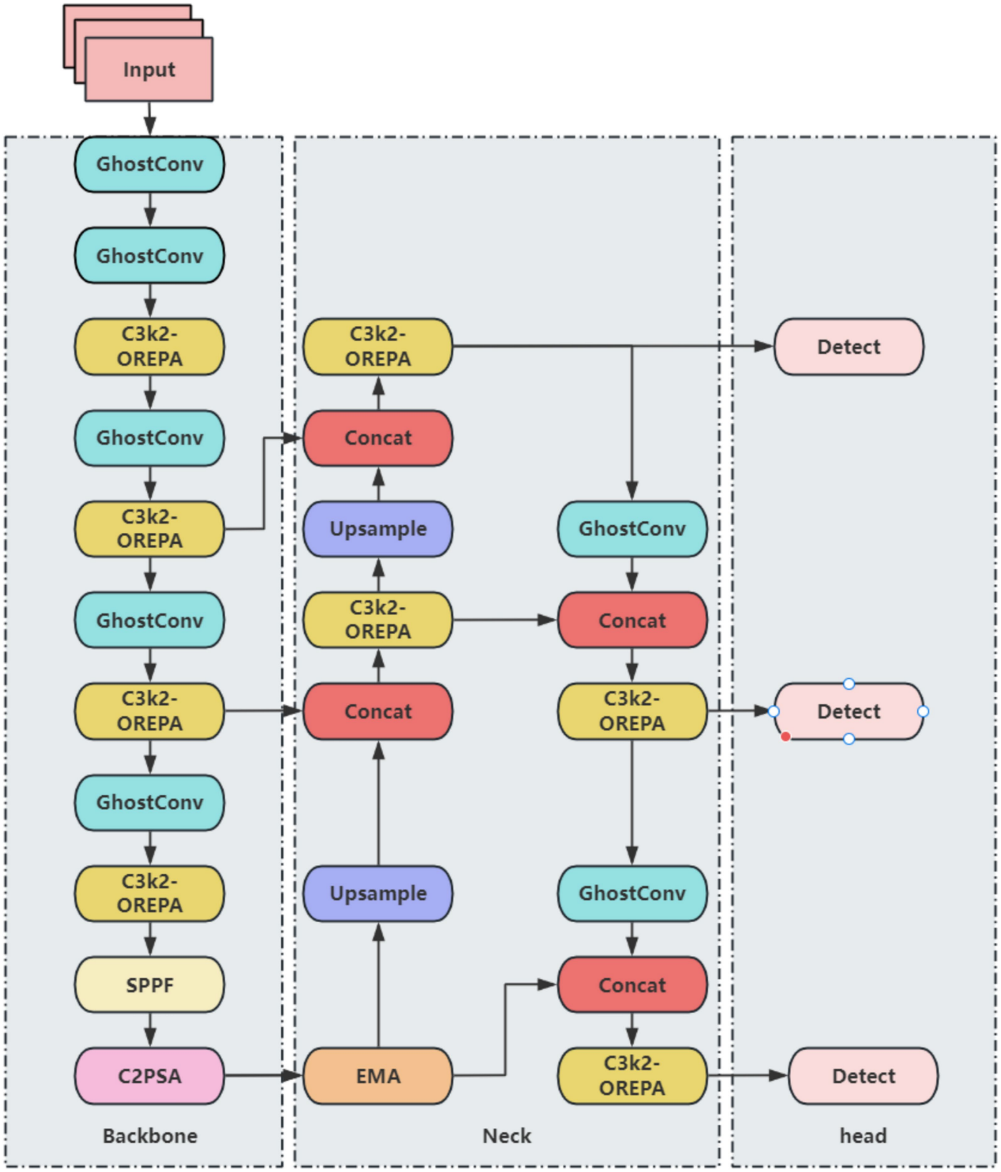
Improved YOLO11 network architecture.

Although YOLO11 demonstrates improvements in both accuracy and speed for brain tumor detection, repeated convolution and pooling operations can compress feature maps, potentially leading to the dilution or loss of small-target features. Additionally, the anisotropic nature of brain tumor structures—including variations in shape, edge, and location—may cause YOLO11 to be biased toward negative predictions. To address these challenges and enhance both detection accuracy and efficiency, a series of improvements are proposed. Direct detection using YOLO11n alone exhibits instability, while employing larger models substantially increases computational and parameter costs. To achieve a balance between accuracy, efficiency, and model lightweighting, several modifications are applied to the YOLO11n framework. The primary improvements include: (1) replacing standard convolutions with the lightweight GhostConv module, (2) integrating OREPA to enhance the C3k2 module, and (3) incorporating the EMA attention mechanism. The resulting improved YOLO11 network architecture is illustrated in Figure 2.

### 2.3 Lightweight GhostConv module

**Figure 3:**
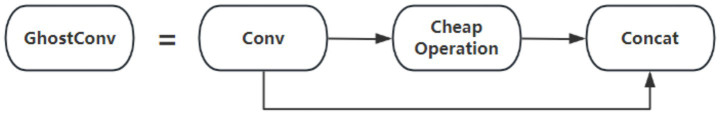
GhostConv module structure.

Traditional feature extraction relies on stacking multiple convolutional kernels, which perform convolutions across all channels of the input feature map, often resulting in redundant computations and high resource consumption. Although conventional convolutions generate rich features, they are computationally intensive and require a large number of parameters. To address the inefficiency caused by traditional convolutions in the YOLO11 architecture, the GhostConv module is introduced as a replacement. GhostConv reduces model parameters, lightens the network, and improves inference speed. The structure of the GhostConv module is illustrated in Figure 3. In GhostConv, a portion of the feature map is initially generated using a standard convolution. Subsequently, a series of linear transformations are applied to this subset of features to produce additional feature maps. These original and transformed feature maps are then concatenated to form the final output. By reducing the learning burden on non-critical features and generating richer representations efficiently, GhostConv decreases both the number of parameters and computational load, thereby enhancing the speed and effectiveness of YOLO11 for brain tumor detection and classification.

### 2.4 Improvement of the C3k2 module

**Figure 4:**
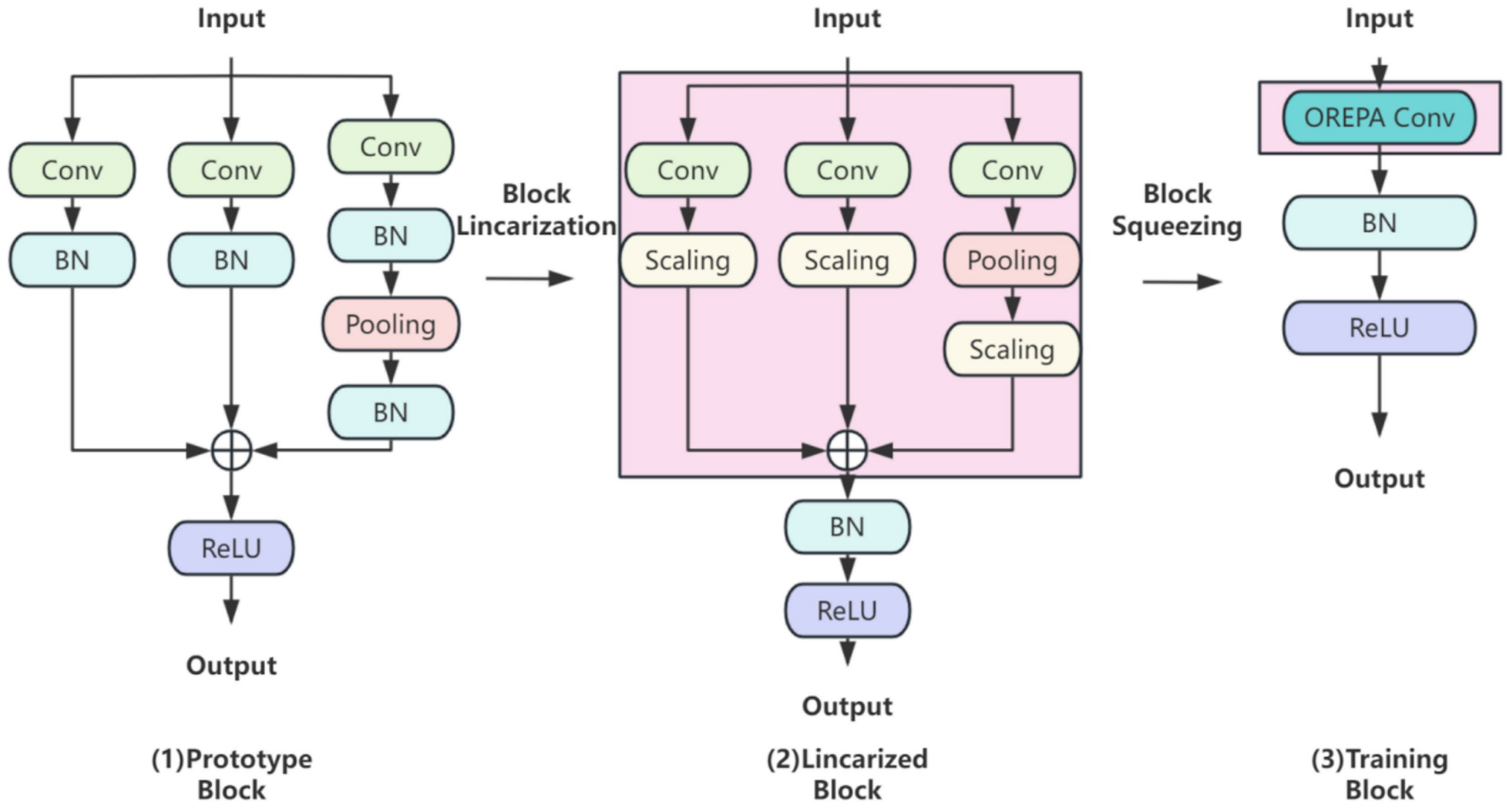
OREPA basic flow.

OREPA (Online Convolutional Reparameterization) enhances training efficiency through two main phases (Figure 4): (1) block linearization, which replaces nonlinear normalization layers with learnable linear scaling layers to reduce computational complexity, and (2) block compression, which merges multi-branch structures into a single convolutional layer using kernel-equivalent transformation. This approach achieves a dual reduction in training memory usage and computational cost for brain tumor detection, while maintaining a lightweight single-layer architecture during inference. In doing so, OREPA addresses the drawbacks of conventional heavily parameterized models, such as high complexity and resource consumption.

In the block linearization phase, nonlinear normalization layers are removed and replaced with linear scaling layers. While normalization layers are commonly employed to stabilize training and accelerate convergence, their nonlinear nature increases training complexity. By contrast, linear scaling layers preserve the effect of branching diversity during optimization while introducing learnable scaling factors that can be directly merged into convolutional layers. Because of their linear property, these scaling factors can be combined with convolutional operations, thereby reducing both computation and memory overhead. This ensures lightweight and efficient model training in brain tumor classification and detection tasks.

In the block compression phase, OREPA consolidates multi-branch and multilayer structures into a single convolutional layer via kernel-equivalent merging. This strategy integrates multiple convolutional layers into one end-to-end operation. Nonlinear BatchNorm (BN) layers are eliminated to further reduce complexity and replaced with linear scaling layers. To stabilize training, a single BN layer is added after branch merging to ensure consistency, as the added BN layer is linearly equivalent and can be merged during inference.

**Figure 5:**
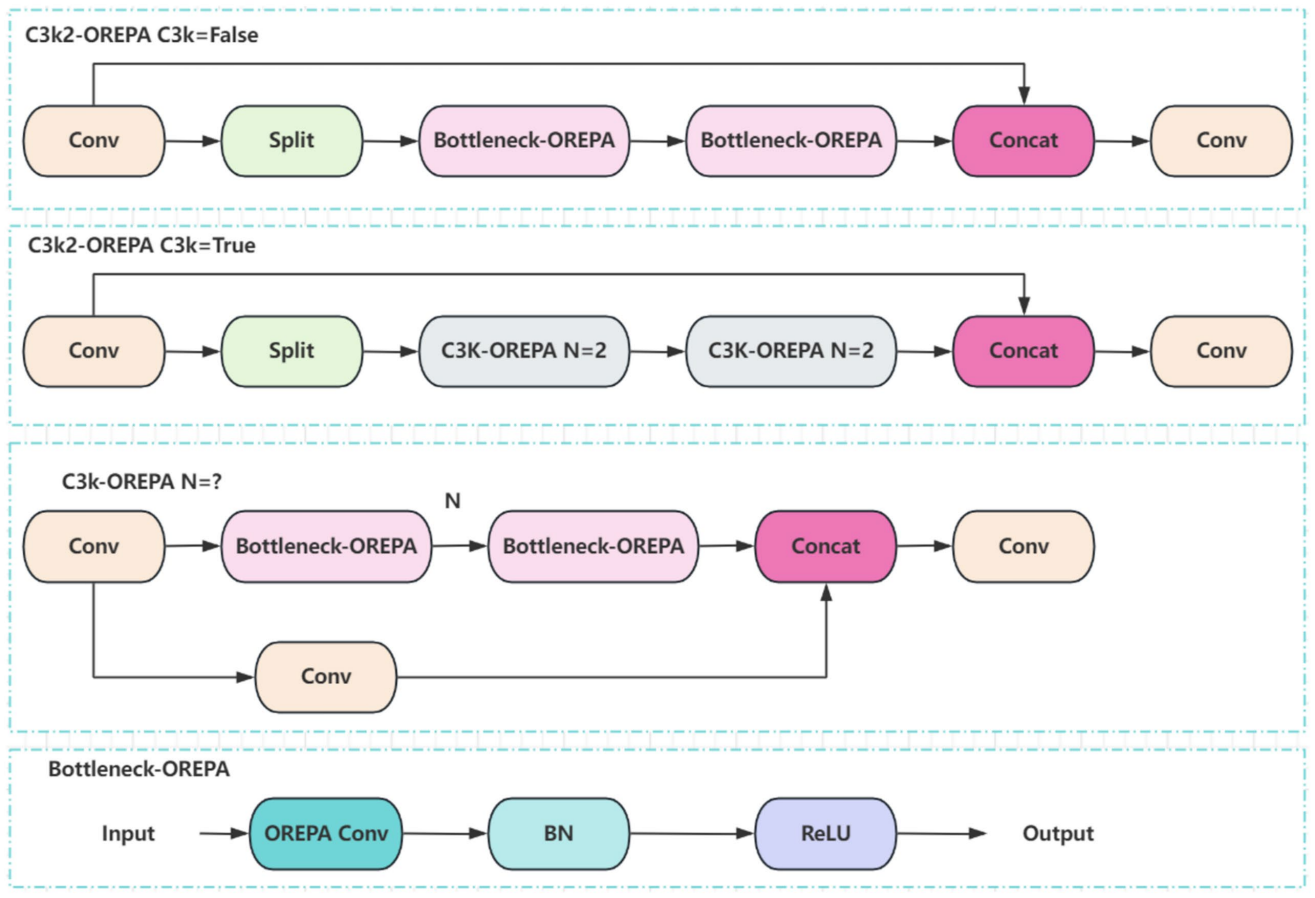
Structure of the C3k2-OREPA model.

The C3k2 module in YOLO11 is an enhanced design derived from the traditional C3 module. By combining variable convolution kernels (e.g., 3 × 3, 5 × 5) with channel separation strategies, it strengthens feature extraction for complex scenes and deep learning tasks. To integrate OREPA into YOLO11, the Bottleneck module within C3k2 is optimized. Specifically, nonlinear BN layers are removed and replaced with linear scaling layers, ensuring diverse optimization directions, while a stabilizing BN layer is added after branch merging. Using the compression principle, the scaling layers are merged into the OREPA structure, forming the Bottleneck-OREPA module. This optimized bottleneck is then embedded into the C3k2 module, creating the C3k2-OREPA module (Figure 5). Replacing the original C3k2 with C3k2-OREPA effectively reduces parameters and computational complexity while preserving strong feature extraction capability. This modification enables YOLO11 to maintain high performance in real-time object detection tasks, while also making the model more suitable for deployment in low-power edge computing environments.

### 2.5 EMA attention mechanism

**Figure 6:**
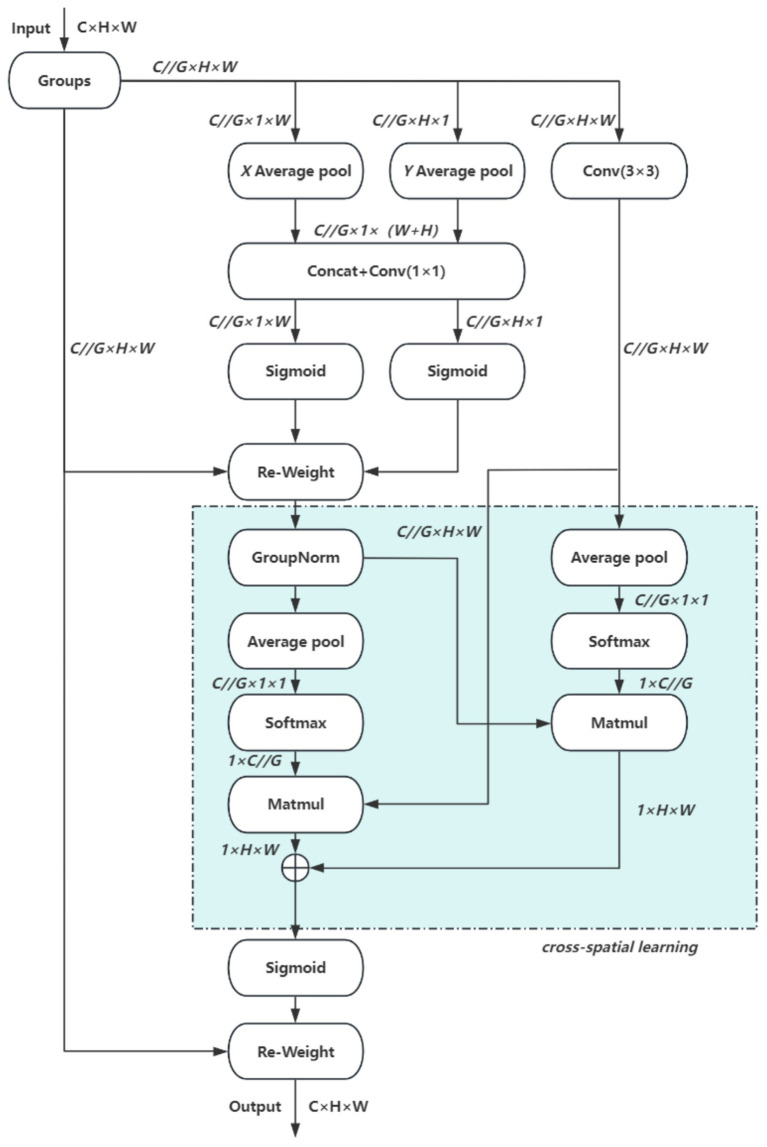
EMA network.

In this study, the EMA (Efficient Multiscale Attention) module is integrated into the YOLO11 architecture. EMA is a lightweight attention mechanism that employs channel grouping to preserve essential information while reducing computational cost. It further encodes global features to recalibrate channel weights and captures pixel-level dependencies through cross-dimensional interactions, making it particularly effective for computer vision tasks. Owing to these properties, EMA achieves both efficiency and flexibility, substantially enhancing model performance without increasing computational overhead. The overall structure of EMA is illustrated in Figure 6.

The EMA module comprises three parallel paths, organized into two types of branches: two 1 × 1 convolutional branches and one 3 × 3 convolutional branch. These branches extract attention weights from feature groups through different strategies. The first type employs one-dimensional global pooling to capture compact channel-level attention, whereas the second incorporates three-dimensional global pooling to enhance feature representation. Initially, a two-dimensional global average pooling operation is applied to the output of the 1 × 1 branch to obtain spatial global features. To enable dimensional alignment during subsequent channel fusion, the smallest branch output is reshaped to a compatible form. The feature representations from the parallel paths are then combined via matrix dot product to generate the first spatial attention map. Subsequently, two-dimensional global average pooling is also applied to the 3 × 3 branch output to acquire global spatial context information. Before activating the channel attention mechanism, the corresponding 1 × 1 branch outputs are adjusted to a consistent dimensional format, enabling pairing with the 3 × 3 branch and resulting in a second spatial attention map that preserves precise spatial location details. Finally, after the output features of both branches are modulated by the Siqmoid function, the output feature maps are used to enhance or attenuate the original input features to obtain the final output.

## 3 Materials and methods

### 3.1 Data acquisition

**Figure 7:**
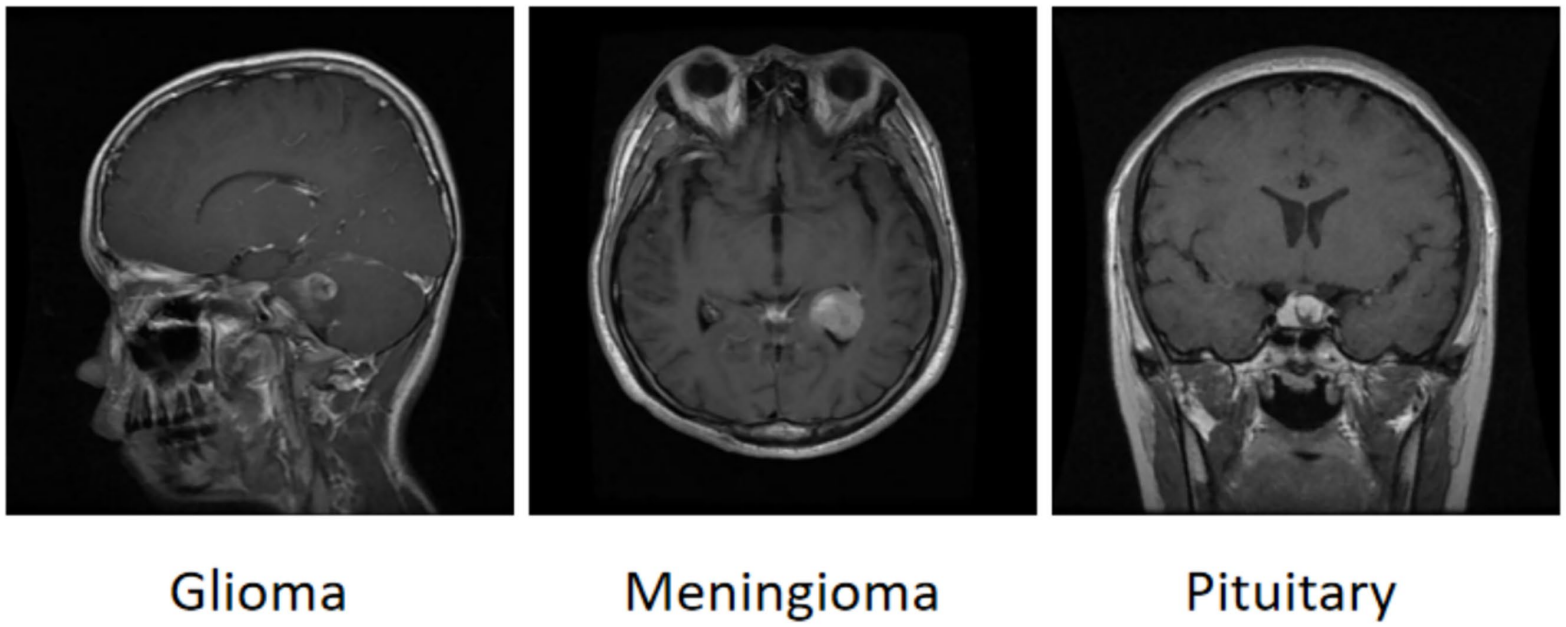
Example of a brain tumor.

**Figure 8:**
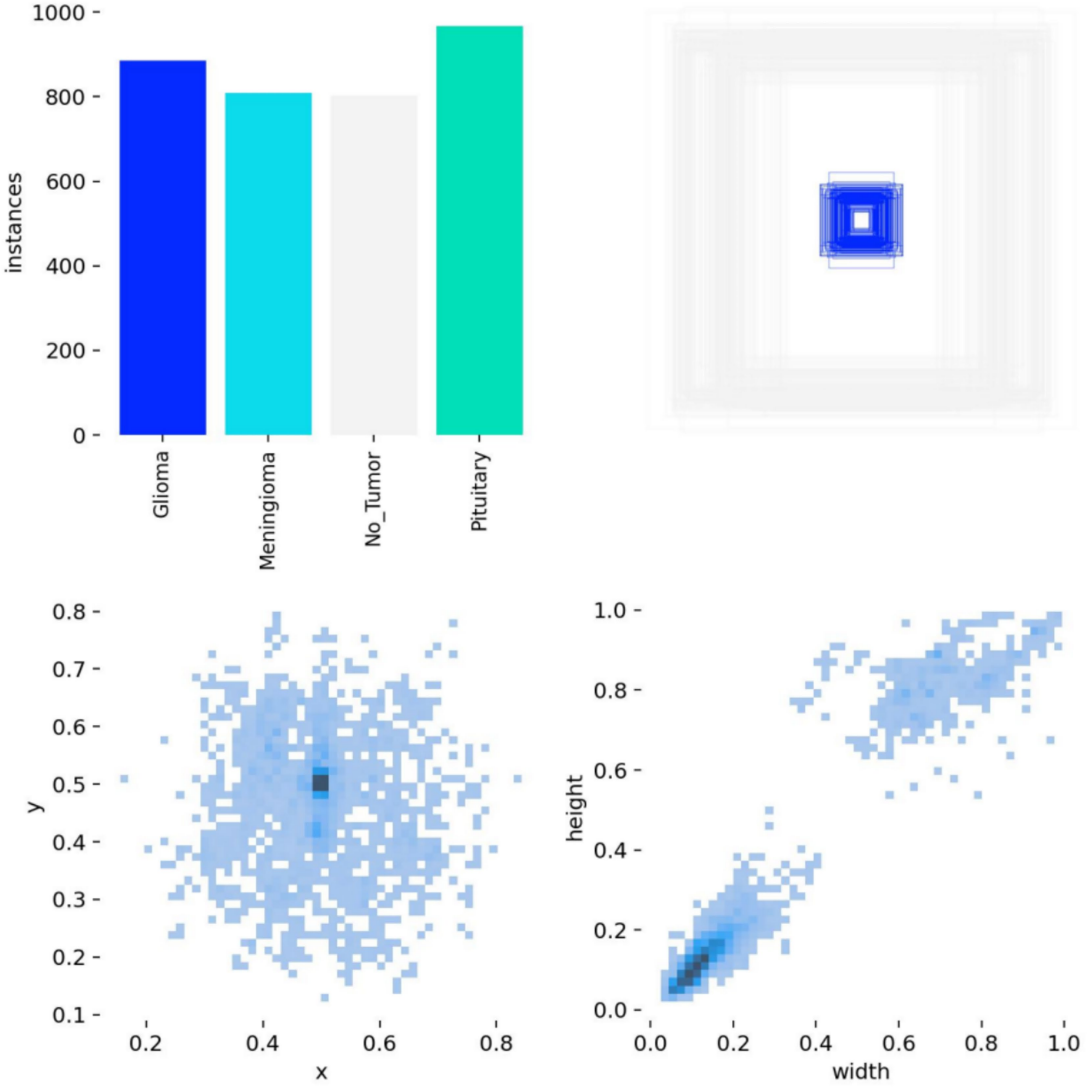
Annotation status in the dataset.

**Figure 9:**
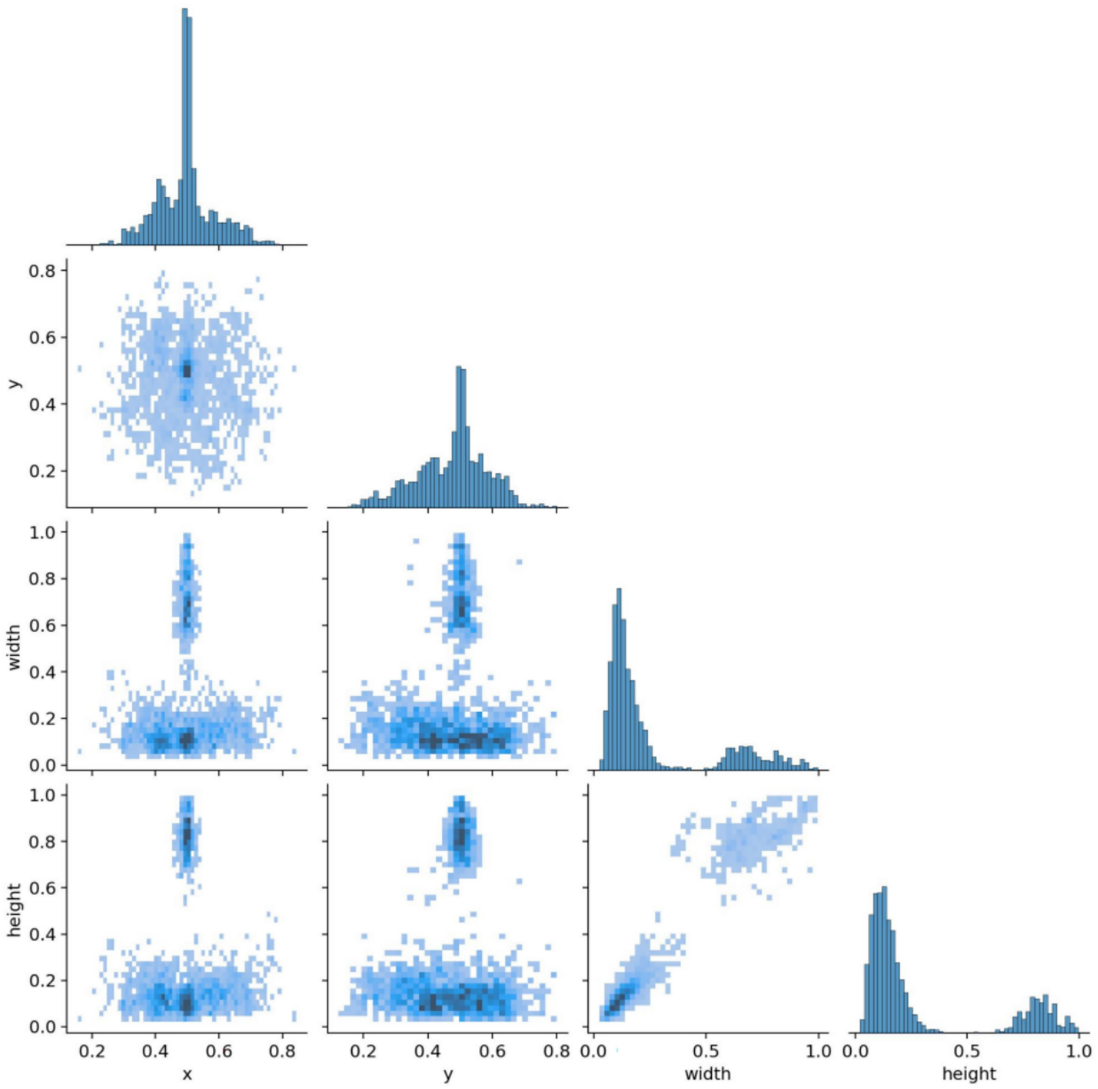
Target correlation graph in the dataset.

The brain tumor classification data used in the experiments were obtained from two publicly available Kaggle datasets. The first dataset (28) was manually filtered to select 4,000 brain MRI images, which were categorized into four groups: glioma, meningioma, pituitary tumor, and no tumor (1,000 images per category). This dataset, referred to as Dataset 1, was used for model training and internal testing. The second dataset (29) contained 20 MRI images per category, totaling 80 images, and served as an external test set, referred to as Dataset 2. All images were annotated in advance by two attending radiologists with over 5 years of experience using the Labeling tool, and the annotations were subsequently reviewed by two senior radiologists with more than 10 years of experience to ensure accuracy. Annotation information was stored in txt format, and all images were resized uniformly to 640 × 640 pixels. Dataset 1 was divided into training, validation, and test sets in an 8:1:1 ratio to guarantee both sufficient training samples and reliable evaluation of model performance. Examples of training samples from Dataset 1 are presented in Figure 7, while the annotation distribution and inter-category correlations are illustrated in Figures 8, 9.

### 3.2 Experimental parameters

The experiment uses windows operating system, CPU model is 12th Gen Intel(R) Core(TM) i5-12400F, GPU model is NVIDIA GeForce GTX4060 graphics card, 8G RAM, and the deep learning framework chooses pytorch-2.4.1, cuda-12.4, and python-3.9.21. The model hyperparameters are set as shown in Table 1. The model hyperparameters were set as shown in Table 1.

**Table 1:** Model hyperparameter settings.

Optimizer	Learning rate	Batch	Epoch	IoU
Adaw	0.00125	32	200	0.7

### 3.3 Experimental evaluation index

The evaluation indicators used in this study are shown in Table 2.

**Table 2:** Experimental evaluation index.

Evaluation index	Symbolic
Average Precision	AP
Precision	P
Recall	R
Union	loU
Average mean precision (loU = 0.5)	mAP@50
Average mean precision (loU = 0.5–0.95)	mAP@50–95
Number of model parameters	Params
Floating point operation	FLOPS

The formula is as follows:

$$P=\frac{N_{\mathrm{TP}}}{N_{\mathrm{TP}}+N_{\mathrm{FP}}}\times 100\%$$

$$R=\frac{N_{\mathrm{TP}}}{N_{\mathrm{TP}}+N_{\mathrm{FN}}}\times 100\%$$

$$\mathrm{mAP}=\frac{1}{n}\sum_{i=1}^{n}{\mathrm{AP}}_{i}$$

$$\mathrm{AP}=\int_{0}^{1}P(r)\mathrm{dr}$$

**Figure 10:**
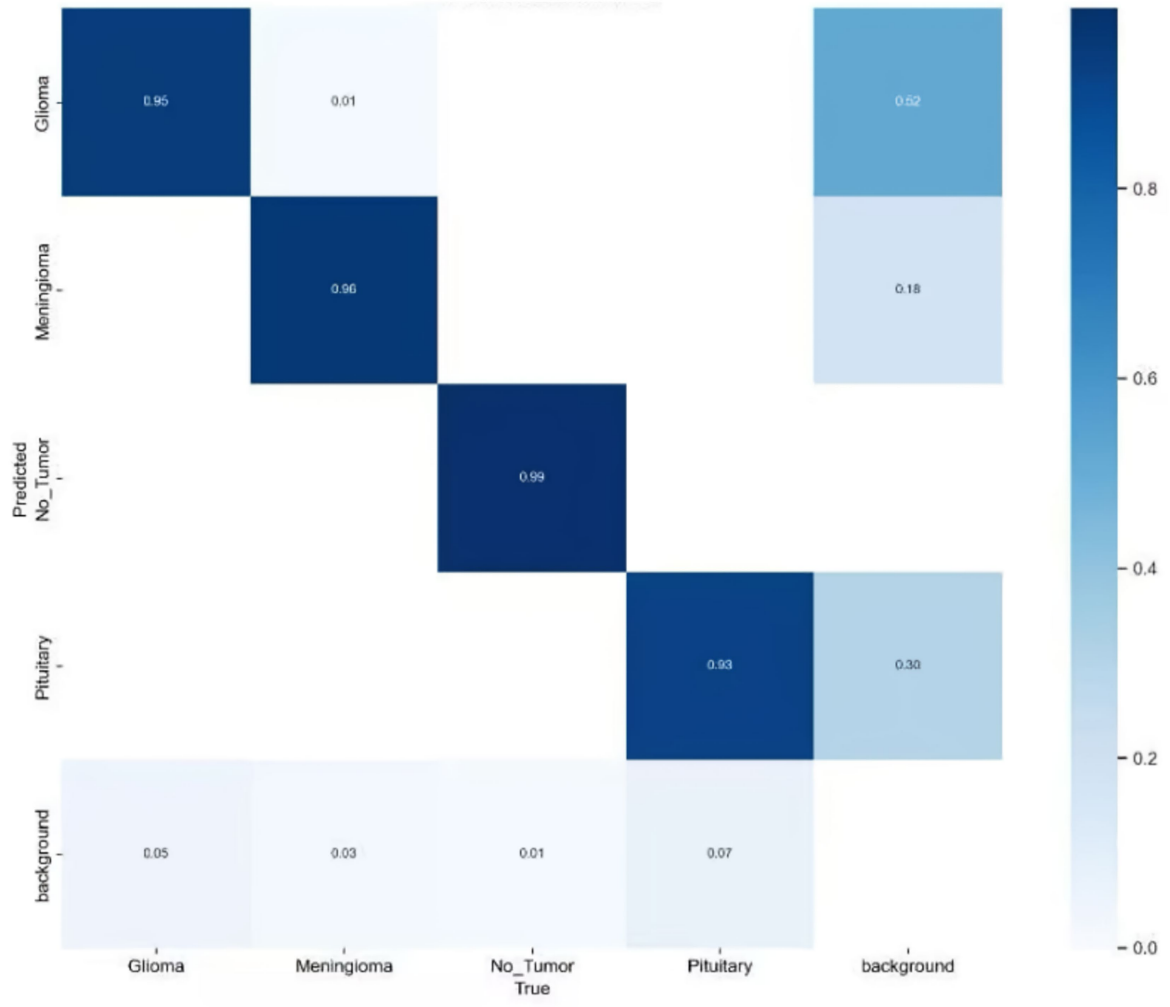
Improved confusion matrix for various types of brain tumors.

**Figure 11:**
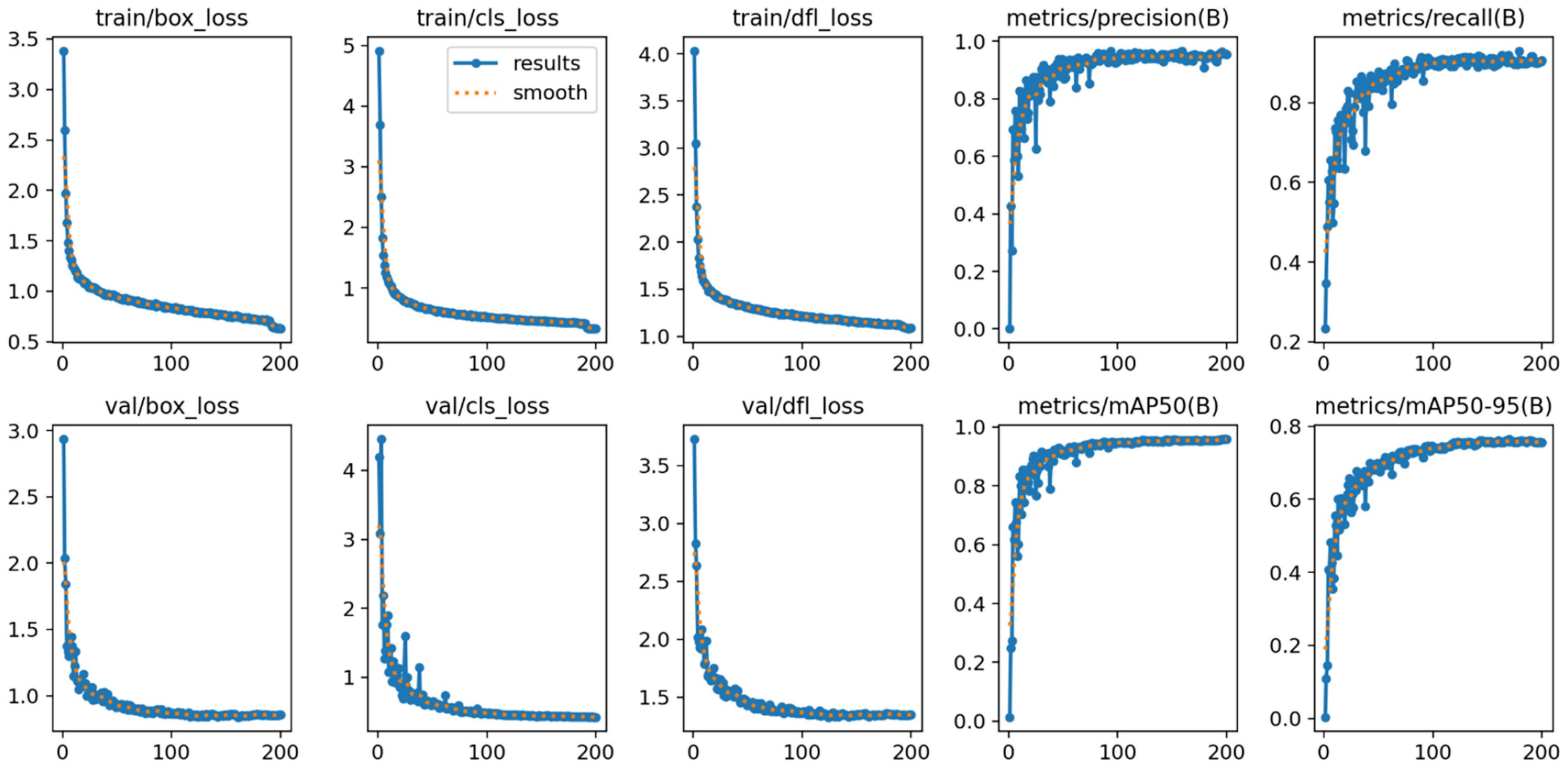
Improved YOLO11n model training curves.

Here, TP denotes the number of true positive cases, FP represents the number of false positive cases, and FN indicates the number of missed detections (false negatives). The variable n refers to the total number of samples, and N denotes the number of categories. In this study, mAP@50 was selected as the primary evaluation index because it emphasizes that moderately overlapping detection is crucial for clinical localization and biopsy guidance, unlike mAP@50–95 which is stricter and may be overly strict for some detections. To comprehensively assess the effectiveness of the proposed model in brain tumor MRI detection, additional performance indicators were introduced, including Precision (P), Recall (R), the number of model parameters (Params), and floating-point operations (FLOPs). These metrics allow for a balanced evaluation of both detection accuracy and computational efficiency. The improved confusion matrix for each tumor category is presented in Figure 10, while the training curve of the improved model is illustrated in Figure 11.

### 3.4 Ablation experiment

In order to verify the effectiveness of each improvement strategy, a systematic ablation experiment was conducted based on YOLO11n, and the experimental results are shown in Table 3. The experimental results show that each improvement module significantly improves the detection performance of the model on the target.

**Table 3:** Ablation experiment.

GhostConv	C3k2-OREPA	EMA	P(%)	R(/%)	mAP_50(_%)	mAP_50-95(_%)	GFLOPS
×	×	×	96.2	88.6	95.1	76.8	6.4
√	×	×	95.8	89.2	95.2	77.1	5.5
×	√	×	95.9	90.7	95.5	77.9	5.7
×	×	√	96.1	92.2	96.5	78.4	6.4
√	√	×	96.0	91.1	95.5	78.1	4.9
√	×	√	96.1	92.5	96.6	78.7	5.9
×	√	√	96.3	92.9	96.8	80.2	5.7
√	√	√	96.4	93.8	97.2	80.7	4.8

**Figure 12:**
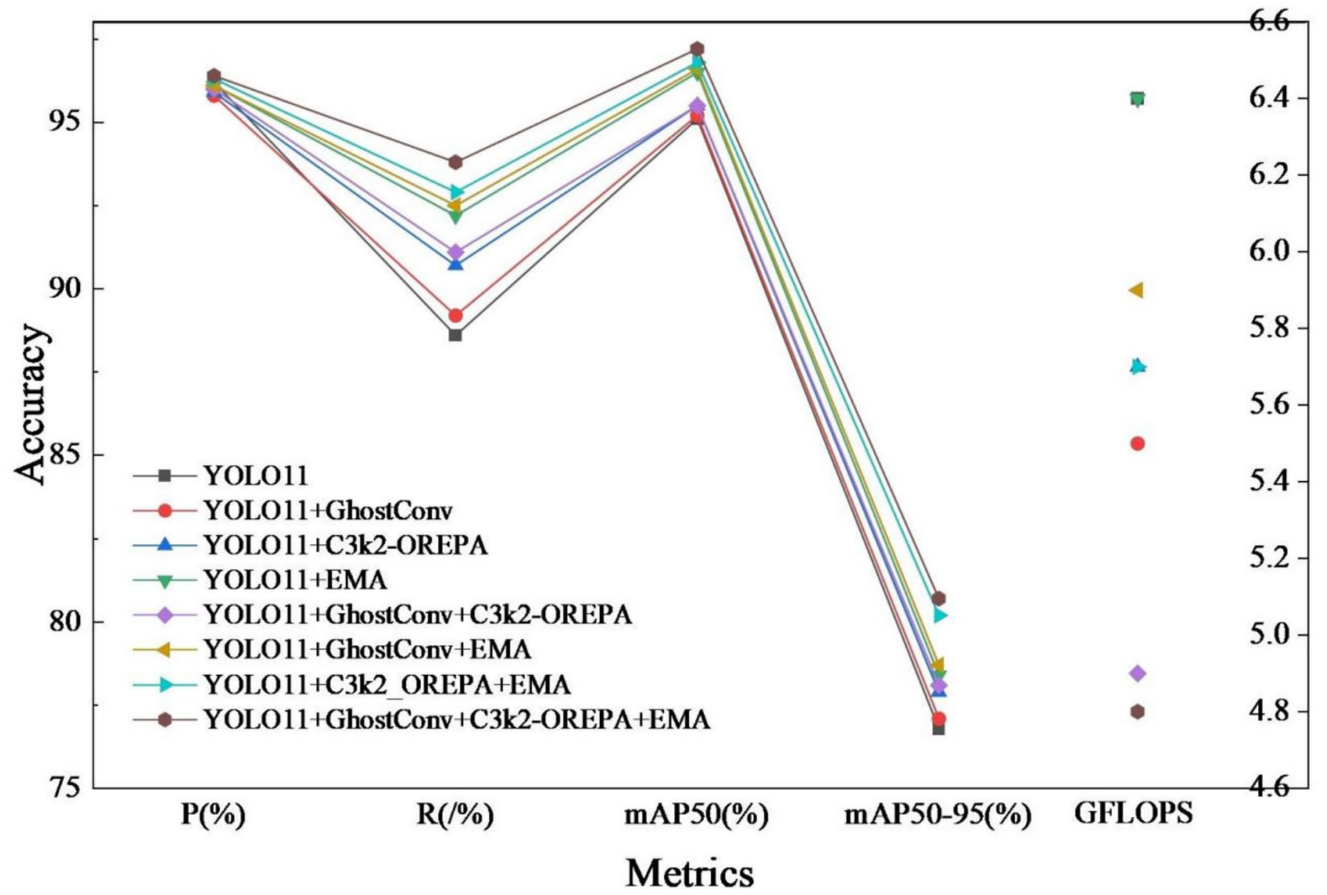
Ablation experiment comparison.

The comparative results of the ablation experiment are shown in Figure 12. As can be seen from the figure, the performance data of the baseline model combined with various improvements.

### 3.5 YOLO series comparison experiment

In order to highlight the performance of the improved algorithm in this paper, other algorithms of the YOLO series of the same magnitude as the YOLO11n model are selected for comparison tests under the same experimental environment, and the obtained experimental results are shown in Table 4. According to the data in the table, in terms of precision, mAP50 as a key indicator for evaluating the comprehensive performance of the model, the mAP50 value of the improved algorithm reaches the highest among the comparative algorithms, 97.2%, which is an increase of 2.1 percentage points on the basis of the original model, and the recall rate of this paper’s algorithm reaches 93.8%, which is the same as the highest value of all the comparative models, and it is a rise of 2.2 percentage points compared with the original model. This paper’s algorithm also reduces the number of parameters and computational complexity while improving precision, reducing the number of parameters by 0.47 M and the complexity by 1.5 GFLOPS compared with the original model.

**Table 4:** Results of comparison experiment.

Model	P(%)	R(%)	mAP_50(_%)	Params(M)	GFLOPS
YOLOv5s	94.8	89.5	93.9	2.18	5.8
YOLOv8n	95.5	90.4	94.7	2.68	6.8
YOLOv10n	95.2	89.2	94.4	2.27	6.5
YOLO11n	95.9	91.6	95.1	2.58	6.4
Ours	96.4	93.8	97.2	2.11	4.8

### 3.6 Analysis of results

**Figure 13:**
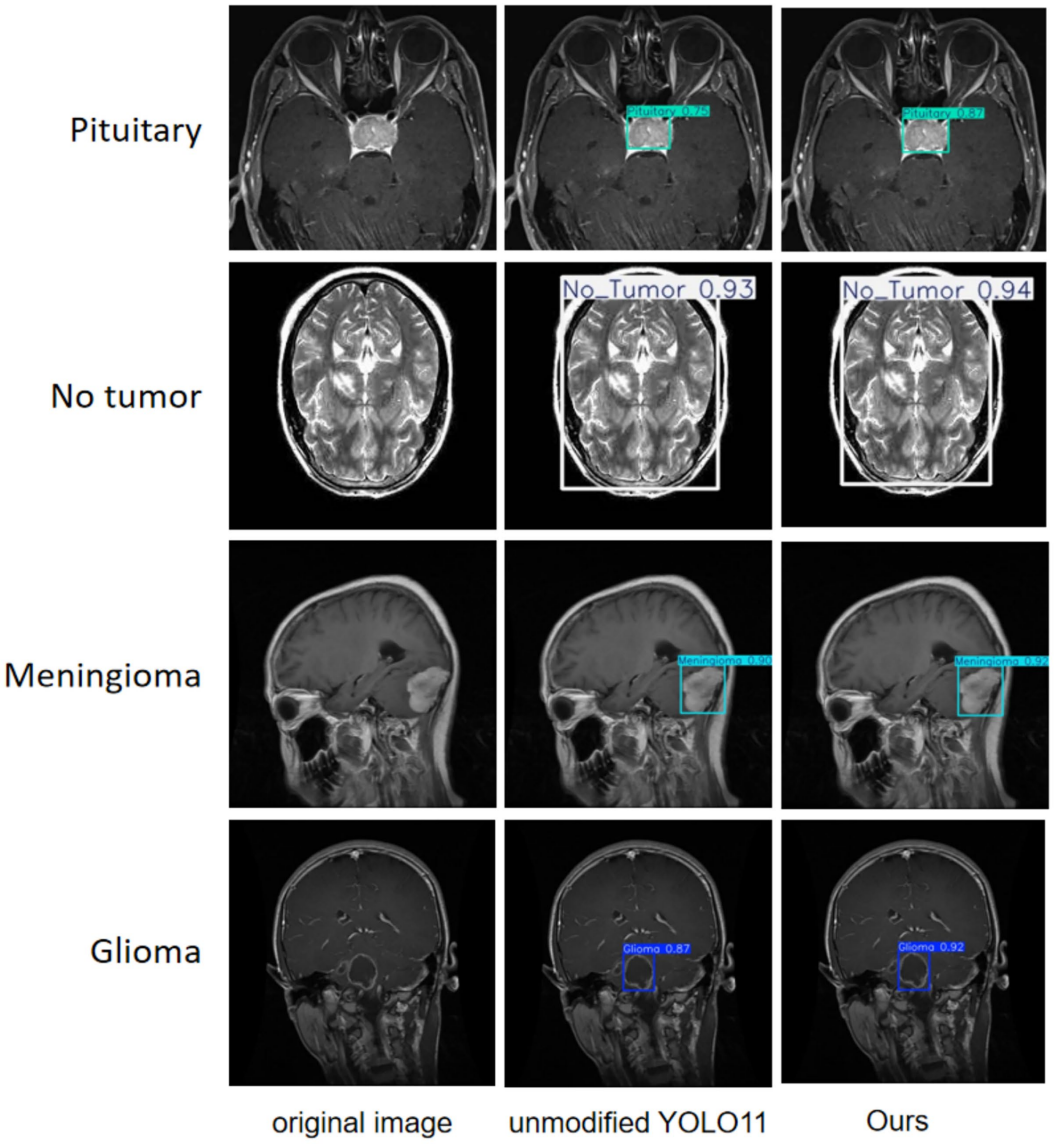
Classification detection results of YOLO model before and after improvement on internal test set.

**Figure 14:**
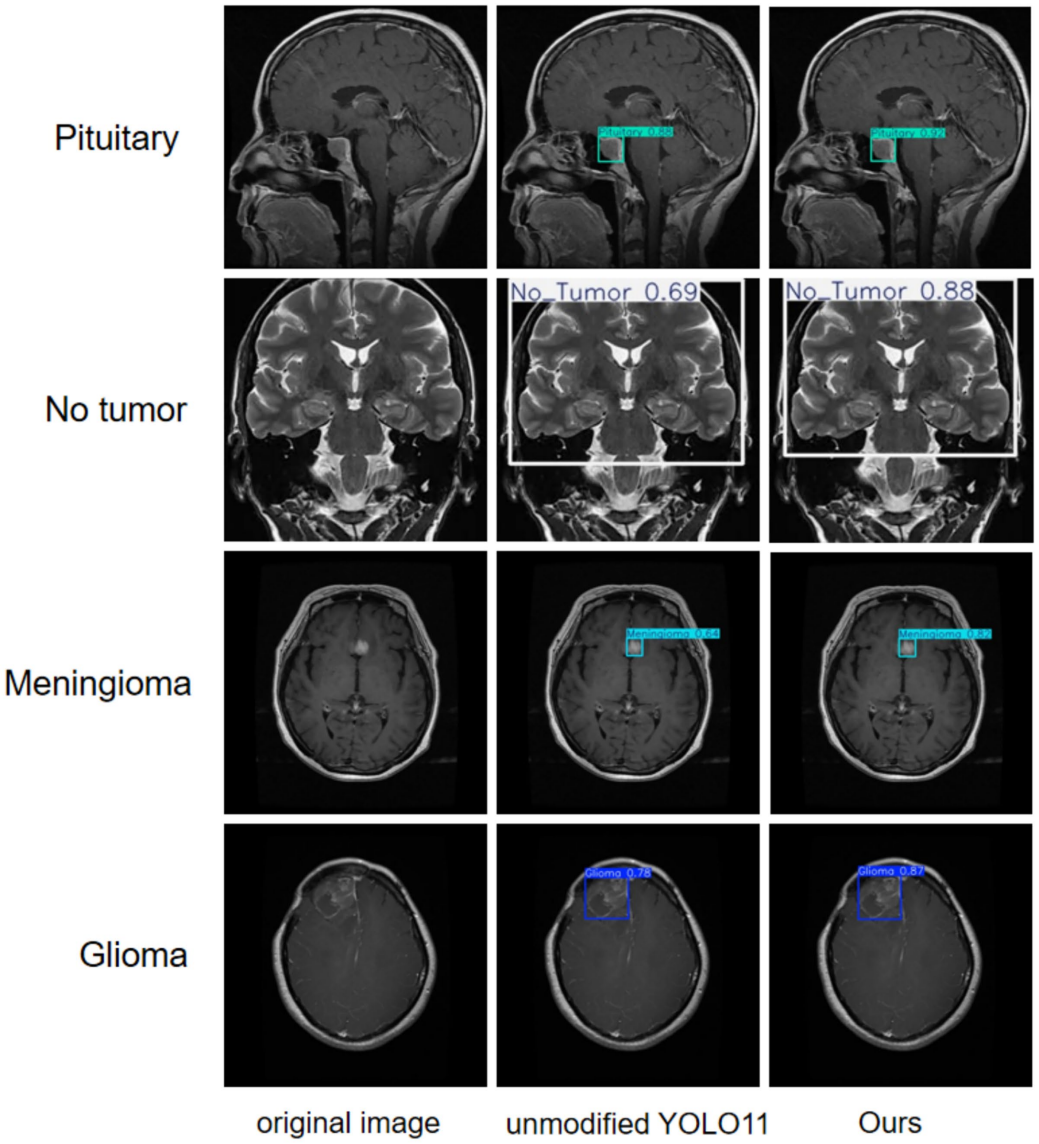
Classification detection results of YOLO model before and after improvement on external test set.

A more intuitive view of the effects before and after the improvement can be seen in the original and detection result plots randomly selected from the internal and external test sets, see Figures 13, 14. Each line compares the effects of a brain tumor type before and after detection and before and after the model improvement, with the original plot, the original YOLO11n detection result plot, and the improved YOLO11n detection result plot, in order from left to right. The text on the left side above the target box in the detection result plots is the brain tumor type, and the number on the right side is the detection confidence level. Column 3 of both Figures 13, 14 show that the improved model can detect gliomas, meningiomas, pituitary tumors, and no tumors (entire brain region) with high confidence. Table 5 shows the quantitative results of the detection performance for various categories of the improved model in the external test set.

**Table 5:** Improved model performance on various categories in the external test set.

Labels	P(%)	R(%)	mAP_50_(%)
Glioma	96.1	93.2	97.0
Meningioma	96.8	94.0	97.5
No tumor	96.9	93.5	97.6
Pituitary	95.6	94.5	96.7

## 4 Discussion

This study presents an improved YOLO11n algorithm that incorporates a lightweight GhostConv module, a C3k2 module optimized via OREPA, and an EMA mechanism. Empirical results indicate that these integrated improvements substantially increase both detection accuracy and computational efficiency in brain tumor MRI image analysis. The revised model achieves a mean average precision (mAP@50) of 97.2% and a recall rate of 93.8%. This high mAP@50 value underscores the model’s exceptional classification precision, which is essential for accurate and reliable clinical diagnosis. Furthermore, the enhanced strategy not only achieves a 2.1% increase in detection accuracy compared to the original model, but also reduces floating-point operations (GFLOPS) by 25% to 4.8, thereby facilitating real-time clinical diagnostics and deployment on edge devices.

The application of the improved YOLO11n model in MRI-based brain tumor detection markedly enhances identification and classification accuracy, thereby supporting the development of personalized treatment strategies. This model serves as a valuable tool for aiding clinical diagnosis and prognostic assessment. Its adoption leads to greater efficiency in medical decision-making and streamlines the diagnostic and therapeutic workflow. With the continuous development of deep learning and artificial intelligence, the invention and integration of more new modules in the future can continuously improve the accuracy, automation level, and real-time performance of models for brain tumor detection. Moreover, the lightweight characteristics of models have enhanced their deployability on edge computing devices, promoting the widespread application of intelligent diagnostic systems in various clinical environments (30, 31).

In the field of deep learning, various models including CNNs (Convolutional Neural Networks)) and ViT (Vision Transformer) have demonstrated significant potential in brain tumor image analysis (32, 33). CNNs, with their powerful local feature extraction capability and translation invariance, have been used in medical image processing achieved a wide range of applications (34). However, CNNs models usually suffer from high computational complexity, high requirements on the amount of training data and tend to overfit on small-sample medical datasets, as well as being sensitive to network architecture design and hyper-parameter tuning (35). In addition, its inherent local receptive field characteristics may limit the effective modeling of image global contextual information, which may become a bottleneck when dealing with brain tumor MRI images with complex spatial dependencies. In contrast, ViT is able to capture long-range dependencies and global contextual information through the self-attention mechanism, which provides a new perspective for understanding the overall structure of images. However, it relies on large-scale pre-training data to fully utilize its advantages, and is prone to underperform or even overfit on relatively limited medical image datasets (36). Meanwhile, ViT models usually have a large number of parameters, significantly higher computational overhead than lightweight CNNs with the same performance, slower inference speed, and their training process is complex and difficult to optimize, which poses substantial challenges for deployment in clinical real-time diagnosis scenarios and resource-constrained devices.

YOLO11n demonstrates significant advantages in real-time brain tumor MRI image detection. As a new generation of end-to-end target detection network optimized for lightweight scenarios, YOLO11n inherits the core features of the YOLO series: it can efficiently predict the target bounding box coordinates and class information directly from the input image, providing good generalization capability and migration potential. The model enhances multi-scale feature extraction by dynamically adjusting the convolutional kernel size and channel strategy through its innovative C3K2 module; optimizes feature fusion and gradient propagation by adopting an improved CSPNet design and PAN structure combined with the C2PSA module; and introduces the depth separable convolution (DWConv) in the classification branch, which effectively reduces the number of parameters and redundancy computation. These designs enable YOLO11n to achieve an excellent balance between speed and accuracy, making it ideal for clinical diagnostic scenarios that require fast responses. In particular, its lightweight architectural design significantly reduces the demand for computational resources, and the model not only runs smoothly on high-performance GPUs, but also has the potential to be deployed to edge computing devices or less configurable hardware environments (e.g., partial CPUs or mobile platforms), which greatly expands its scope of application in diversified healthcare environments (e.g., primary healthcare centers, mobile terminals). In addition, it adopts a consistent dual-allocation strategy to directly generate the final detection frame, eliminating the non-maximum suppression (NMS) post-processing step, further reducing computational overhead and improving real-time performance.

In brain tumor detection tasks, high recall is of primary clinical importance to ensure that no suspicious tumor regions are missed. Even if the model possesses high precision, a low recall may result in certain tiny or ambiguous tumor lesions being overlooked, with serious consequences. However, simultaneous enhancement of precision is equally crucial, as it is directly related to the accuracy of target localization and classification, which not only significantly reduces the possibility of misclassifying non-tumor normal tissues as tumor categories and reduces unnecessary patient anxiety and invasive examinations for non-tumor lesions, but also provides clinicians with a reliable AI-assisted basis for quickly and accurately identifying tumor subtypes. This high-precision classification result is a critical first step in the development of personalized treatment plans, it directly determines whether the patient should be prioritized for surgical resection, radiotherapy, chemotherapy, or conservative observation and follow-up, which helps to direct the patient to the most appropriate diagnostic and therapeutic pathway at the early stage of the disease, and avoids delays in treatment or inappropriate choices of treatment due to misdiagnosis or ambiguity in classification. At the same time, clear information on the tumor category also provides important reference information for the subsequent estimation of the difficulty of surgery, the need for multidisciplinary collaboration, and the prediction of the sensitivity of radiotherapy, laying the foundation for more detailed diagnostic and therapeutic steps.

However, there are still limitations in this study: the model training relies on a single-source static MRI dataset, which does not cover multi-center, multi-device, and multi-modality images, which may limit its clinical generalization ability; in the face of extremely rare or morphologically atypical tumor subtypes, the robustness of the model needs to be verified by a larger dataset; and the lesions in the dataset are manually annotated, which is subjective and may contain errors. Future research should explore multi-center and multimodal datasets (e.g., MRI, CT, PET) and investigate integration into clinical systems such as PACS, as well as deployment in mobile and intraoperative settings to enhance real-world applicability.

## 5 Conclusion

In this study, several enhancements were introduced to the YOLO11 model to improve brain MRI tumor detection. GhostConv was incorporated into the feature extraction stage to reduce redundant computation and accelerate detection. The C3k2 module was optimized by embedding OREPA, thereby lowering parameter complexity while maintaining efficiency. In addition, the EMA attention mechanism was integrated to strengthen feature representation and better capture the heterogeneity of brain tumors. Experimental results demonstrated that, compared with the original YOLO11 model, the improved framework substantially reduced computational cost while achieving higher detection accuracy. The optimized model achieved 97.2% mAP@50 with a reduced computational load of 4.8 GFLOPS, confirming its effectiveness in balancing accuracy and efficiency. These advancements have facilitated the translation to intraoperative tools, promising to revolutionize the management of brain tumors.

## Data Availability

The original contributions presented in the study are included in the article/supplementary material, further inquiries can be directed to the corresponding author.

## Footnotes

^1^ https://github.com/ultralytics/ultralytics
